# Co-crystallization of 3,5-di­nitro­benzoic acid with two anti­psychotic agents: a simple 1:1 salt with trihexyphenidyl and a 1:2 acid salt containing a very short O—H⋯O hydrogen bond with chlorprothixene

**DOI:** 10.1107/S2056989019001385

**Published:** 2019-01-31

**Authors:** Mohammed A. E. Shaibah, Hemmige S. Yathirajan, Ravindranath S. Rathore, Tetsundo Furuya, Tomoyuki Haraguchi, Takashiro Akitsu, Christopher Glidewell

**Affiliations:** a Department of Studies in Chemistry, University of Mysore, Manasagangotri, Mysuru 570 006, India; b Department of Bioinformatics, School of Earth, Biological and Environmental Sciences, Central University of South Bihar, Gaya 824 236, India; c Department of Chemistry, Faculty of Science, Tokyo University of Science, 1-3 Kagurazaka, Shinjuku-ku, Tokyo 162-8601, Japan; d School of Chemistry, University of St Andrews, St Andrews, Fife KY16 9ST, UK

**Keywords:** co-crystallization, acid salts, crystal structure, mol­ecular conformation, hydrogen bonding, supra­molecular assembly

## Abstract

Co-crystallization of racemic 1-cyclo­hexyl-1-phenyl-3-(piperidin-1-yl)propan-1-ol (trihexyphenid­yl) with 3,5-di­nitro­benzoic acid gives a simple 1:1 salt, namely 1-(3-cyclo­hexyl-3-hy­droxy-3-phenyl­prop­yl)piperidin-1-ium 3,5-di­nitro­benzoate, C_20_H_32_NO^+^·C_7_H_3_N_2_O_6_^−^, (I), whereas a similar co-crystallization using (*Z*)-3-(2-chloro-9*H*-thioxanthen-9-yl)-*N*,*N*-di­methyl­propan-1-amine (chlorprothixene) gives a 1:2 acid salt, namely (*Z*)-3-(2-chloro-9*H*-thioxanthen-9-yl)-*N*,*N*-di­methyl­propan-1-aminium hydrogen bis­(3,5-di­nitro­benzoate), C_18_H_19_ClNS^+^·[H(C_7_H_3_N_2_O_6_)_2_]^−^, (II), the anion of which contains a very short O—H⋯O hydrogen bond, with dimensions O—H = 1.04 (3) Å, H⋯O = 1.41 (3) Å, O⋯O = 2.4197 (15) Å and O—H⋯O = 161 (3)°. In the cation of (I), the cyclo­hexyl and piperidyl rings both adopt chair conformations, whereas in the cation of (II), the central heterocyclic ring adopts a boat conformation, so that the dihedral angle between the two aryl rings is 41.56 (4)°. A combination of O—H⋯O, N—H⋯O and C—H⋯O hydrogen bonds links the ions of (I) into a complex chain of rings, and these chains are linked into sheets by π–π stacking inter­actions between inversion-related pairs of anions. In compound (II), a different combination of O—H⋯O, N—H⋯O and C—H⋯O hydrogen bonds links the ions into sheets. Comparisons are made with some related structures.

## Abstract 2

Co-crystallization of 3,5-di­nitro­benzoic acid with trihexyphenidyl [or 1-cyclo­hexyl-1-phenyl-3-(piperidin-1-yl)propan-1-ol], gives a 1:1 salt (I) but chlorprothixene [or (*Z*)-3-(2-chloro-9*H*-thioxanthen-9-yl)-*N*,*N*-di­methyl­propan-1-amine], gives a 1:2 acid salt (II) containing a very short O—H⋯O hydrogen bond. Multiple hydrogen bonds link the ions in (I) into a complex chain of rings and those in (II) link the ions into a sheet.

## Chemical context   

**Figure 1:**
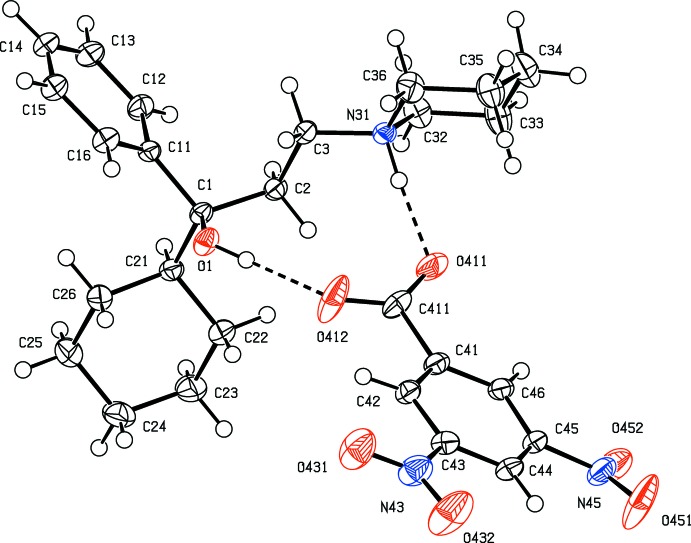
The mol­ecular structure of compound (I), showing the (*S*)-enanti­omer of the cation, the atom-labelling scheme and the hydrogen bonds, drawn as dashed lines, within the selected asymmetric unit. Displacement ellipsoids are drawn at the 50% probability level.

**Figure 2:**
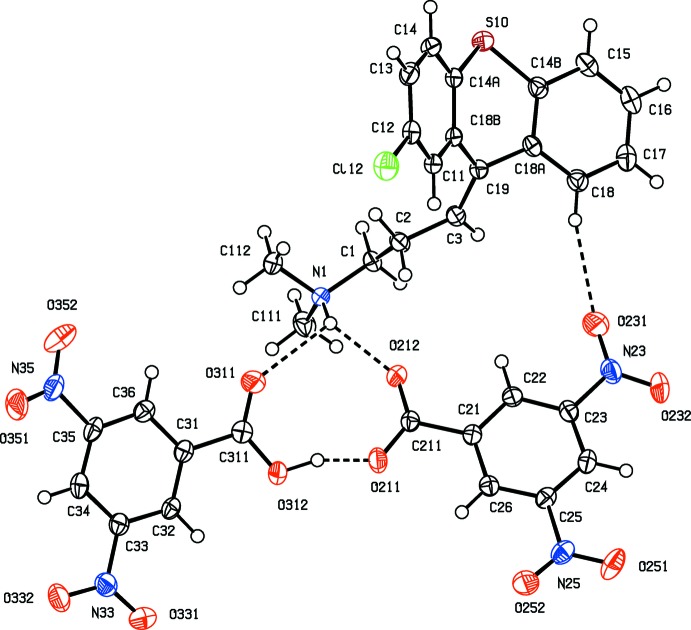
The mol­ecular structure of compound (II) showing the atom-labelling scheme and the hydrogen bonds, drawn as dashed lines, within the selected asymmetric unit. Displacement ellipsoids are drawn at the 50% probability level.

1-Cyclo­hexyl-1-phenyl-3-(piperidin-1-yl)propan-1-ol, trihexy­phenidyl, and 3-(2-chloro-9*H*-thioxanthen-9-yl)-*N*,*N*-di­methyl­propan-1-amine, chlorprothixene, can both be used in the treatment of psychotic depression (Roth *et al.*, 1994 ▸; Seeman & Tallerico, 1998 ▸; Silvestre & Prous, 2005 ▸). In addition, trihexyphenidyl is well established as a treatment for symptomatic relief in cases of Parkinson’s disease (Doshay *et al.*, 1954 ▸). Trihexyphenidyl is generally administered as the hydro­chloride salt but, although the structures have been reported for both neutral trihexyphenidyl (Camerman & Camerman, 1972 ▸) and neutral chlorprothixene (Post *et al.*, 1974 ▸), there are few reported structures for salts derived from either of these two bases, although we note a powder diffraction study of trihexiphenidyl hydro­chloride (Maccaroni *et al.*, 2010 ▸). Accordingly, we have now investigated the salts formed by trihexyphenidyl and chlorprothixene with 3,5-di­nitro­benzoic acid. Crystallization of equimolar mixtures of racemic trihexiphenydine or (*Z*)-chlorprothixene with 3,5-dintro­benzoic acid yielded a simple 1:1 salt in the case of trihexyphenidyl (Fig. 1 ▸), but a 1:2 acid salt in the case of chlorprothixene (Fig. 2 ▸): within fairly wide limits, regardless of the initial stoichiometry of the co-crystallization mixtures, the same products were always obtained.
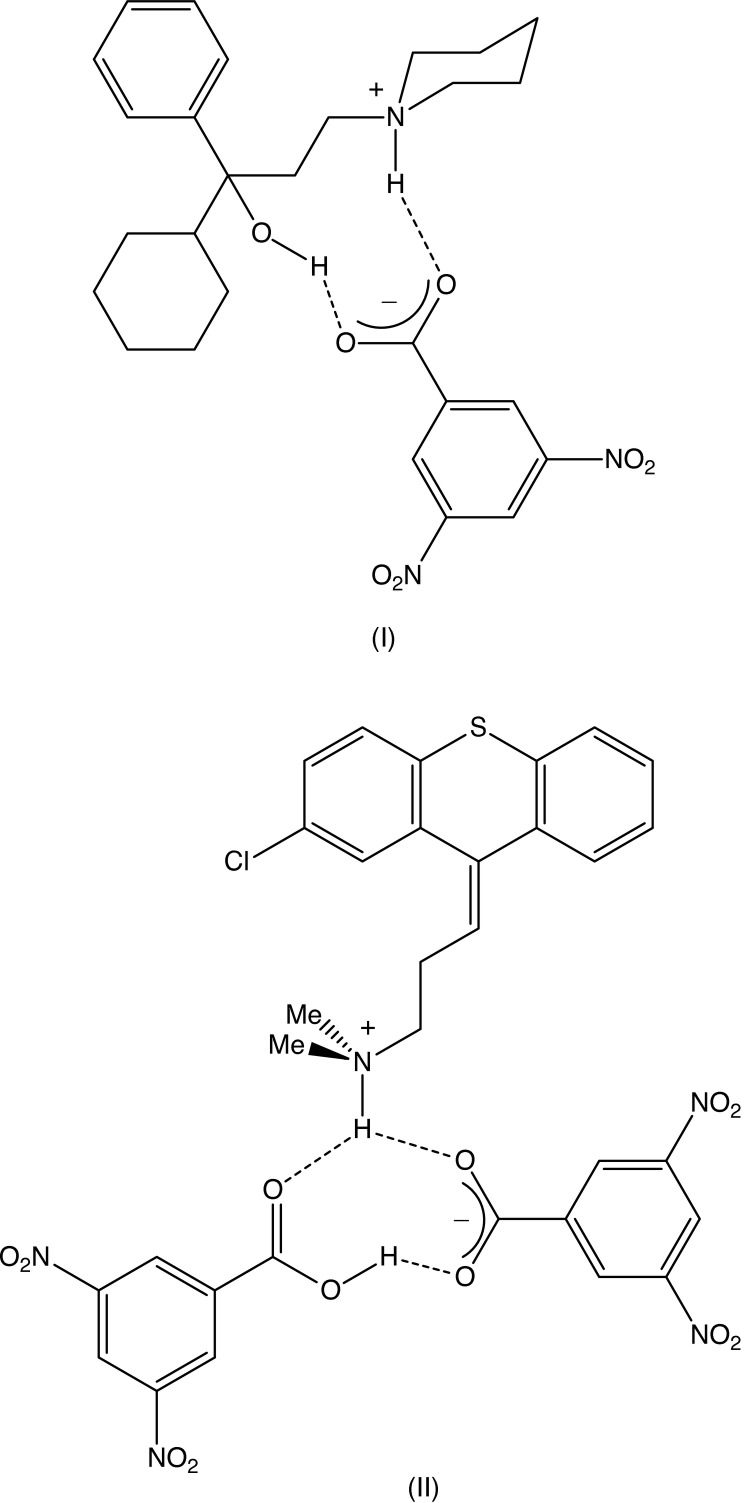


## Structural commentary   

Co-crystallization from a methanol solution containing equimolar qu­anti­ties of racemic trihexyphenidine and 3,5-di­nitro­benzoic acid gave a simple 1:1 salt (I) (Fig. 1 ▸), but a similar crystallization using equimolar qu­anti­ties of (*Z*)-3-(2-chloro-9*H*-thioxanthen-9-yl)-*N*,*N*-di­methyl­propan-1-amine and 3,5-di­nitro­benzoic acid gave an acid salt (II) containing the hydrogen bis­(3,5-di­nitro­benzoate) anion (Fig. 2 ▸). Within this anion, the O—H⋯O hydrogen bond (Table 2 ▸) is very short (Speakman, 1972 ▸; Emsley, 1980 ▸; Gerlt *et al.*, 1997 ▸) but, although it is nearly linear, it is not symmetric as the two independent O—H distances are significantly different (Table 2 ▸).

In the selected asymmetric unit of (I) (Fig. 1 ▸), the ionic components are linked by just two hydrogen bonds, one each of O—H⋯O and N—H⋯O types (Table 1 ▸), to form a compact unit containing an 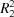
(10) (Etter, 1990 ▸; Etter *et al.*, 1990 ▸; Bernstein *et al.*, 1995 ▸) ring. By contrast, within the selected asymmetric unit of (II), the components are linked not only by the short O—H⋯O hydrogen bond referred to above, but also by a three-centre N—H⋯(O)_2_ hydrogen bond and a two-centre C—H⋯O hydrogen bond (Fig. 2 ▸, Table 2 ▸).

**Figure 3:**
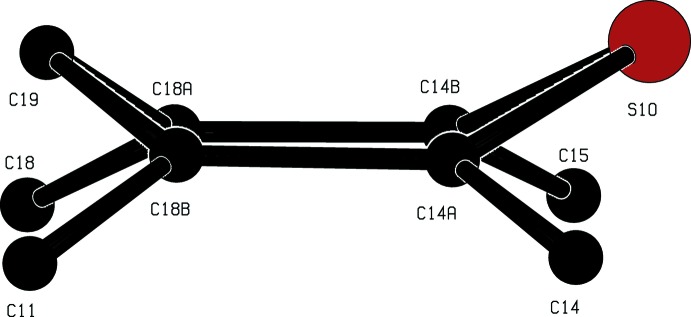
The boat conformation of the thio­pyran ring in compound (II), including all the immediate ring substituent atoms.

The cyclo­hexyl and piperidyl rings in the cation of compound (I) both adopt chair conformations with the sole C-substituent occupying an equatorial site in each case (Fig. 1 ▸). In the cation of compound (II), the dihedral angle between the two aryl rings is 41.56 (4)°, indicating a butterfly conformation for the tricyclic component; the central ring adopts a boat conformation where the atoms C14*A*, C14*B*, C18*A* and C18*B* are coplanar with the atoms C19 and S10, which form the bow and stern of the boat (Fig. 3 ▸), displaced from the plane of the other four ring atoms by 0.456 (2) and 0.541 (2) Å respectively. The ring-puckering parameters calculated for the atom sequence (S10, C14*A*, C18*B*, C19, C18*A*, C14*B*) are *Q* = 0.5721 (12) Å, θ = 86.71 (13)° and φ = 0.53 (14)°: for an idealized boat form the puckering angles are θ = 90.0° and φ = 60*k*°, where *k* represents an integer (Boeyens, 1978 ▸).

In the anion of (I), the nitro group containing atom N43 forms a dihedral angle of only 3.03 (3)° with the adjacent ring, but the other nitro group and the carboxyl­ate group form angles of 21.3 (2) and 20.4 (2)°, respectively. Comparable differences are observed also in the anionic component of (II), where the carboxyl­ate groups form dihedral angles with the adjacent rings of 3.6 (2) and 11.8 (2)°, while the corresponding angles for the four nitro groups range from 3.5 (2) to 18.2 (2)°.

## Supra­molecular features   

**Figure 4:**
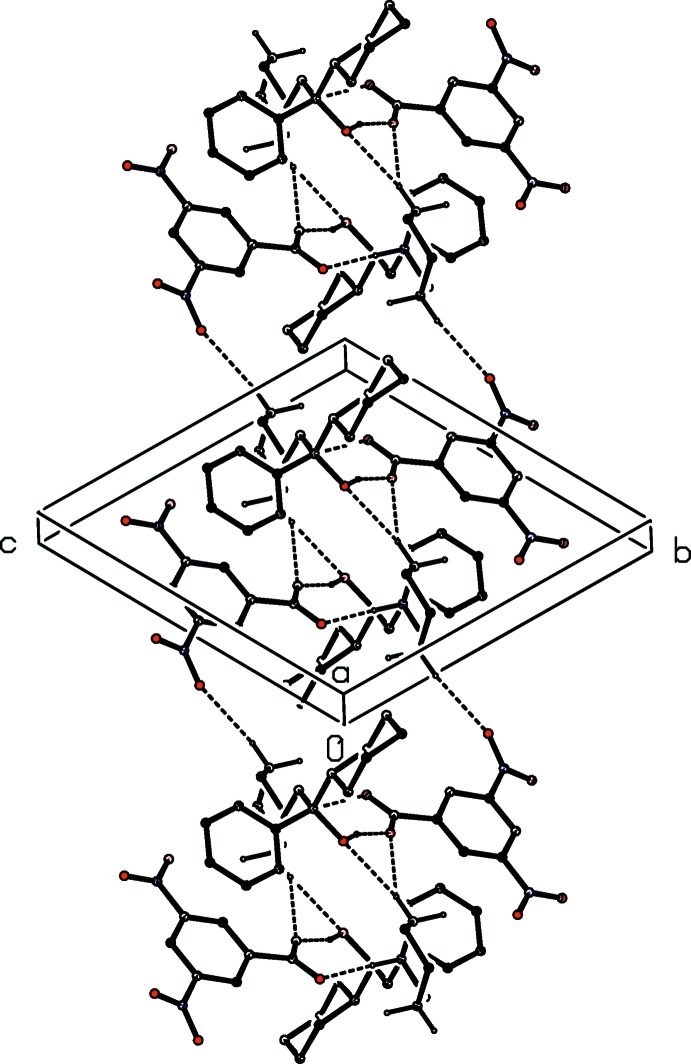
Part of the crystal structure of compound (I) showing the formation of a hydrogen-bonded chain of rings parallel to [011]. Hydrogen bonds are drawn as dashed lines and, for the sake of clarity, the H atoms bonded to the C atoms which are not involved in the motifs shown have been omitted.

**Figure 5:**
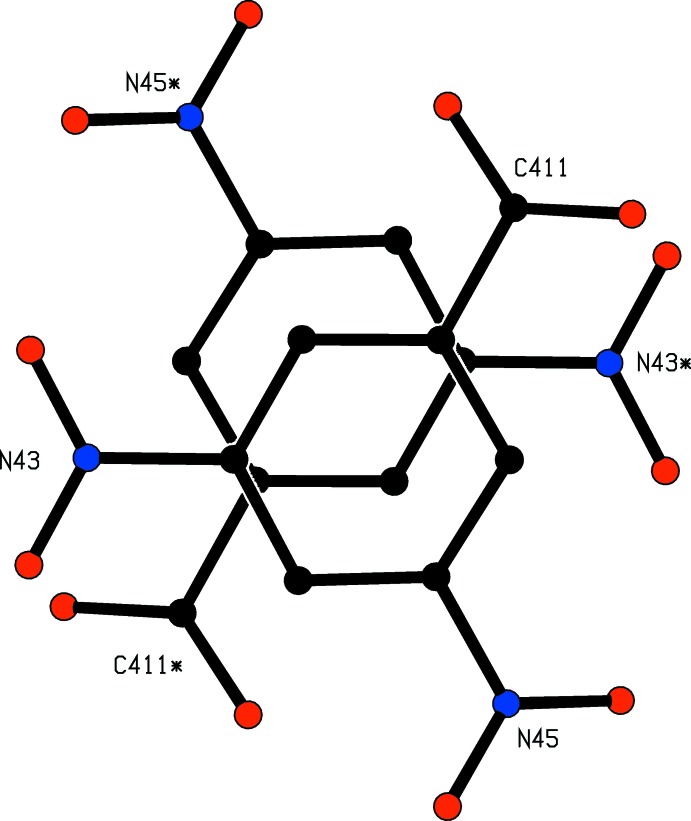
Part of the crystal structure of compound (I) showing the π–π stacking overlap between the anions at (*x*, *y*, *z*) and (1 − *x*, −*y*, 1 − *z*). For the sake of clarity, the unit-cell outline and all of the H atoms have been omitted. The atoms marked with an asterisk (*) are at the symmetry position (1 − *x*, −*y*, 1 − *z*).

In addition to the hydrogen bonds within the selected asymmetric unit of compound (I) (Fig. 1 ▸, Table 1 ▸), the resulting ion-pairs are linked by three independent C—H⋯O hydrogen bonds, which together generate a complex chain structure (Fig. 4 ▸). The hydrogen bond involving atom C33 as the donor links inversion-related pairs of cations and anions to form a four-ion aggregate characterized by an 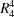
(24) motif. The hydrogen bond involving atom C36 as the donor, by contrast, forms an almost planar three-centre C—H⋯(O)_2_ system, again linking inversion-related ion pairs to form a complex motif in which a central 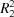
(14) ring containing only cations is concentric with an outer 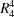
(14) motif involving both cations and anions. The 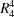
(24) rings are centred at (½, *n*, *n*) and the fourteen-membered rings are centred at (½, *n* + ½, *n* + ½), where *n* represents an integer in each case, so forming a chain of rings running parallel to the [011] direction (Fig. 4 ▸). Chains of this type are linked by a π–π stacking inter­action involving the anions at (*x*, *y*, *z*) and (1 − *x*, −*y*, 1 − *z*). These rings are strictly parallel, with an inter­planar spacing of 3.4413 (6) Å: the ring-centroid separation is 3.5231 (10) Å, corresponding to a ring-centroid offset of 0.755 (2) Å (Fig. 5 ▸), and this inter­action links the hydrogen-bonded chains into a sheet lying parallel to (100).

**Figure 6:**
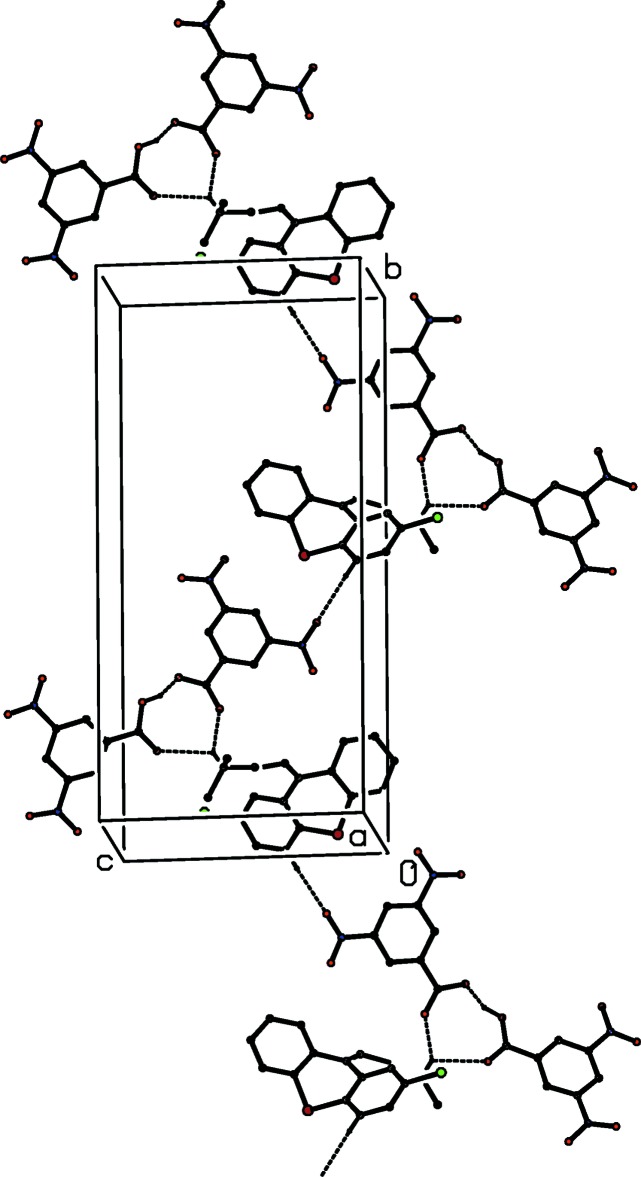
Part of the crystal structure of compound (II) showing the formation of a hydrogen-bonded 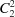
(7) chain running parallel to the [010] direction. Hydrogen bonds are drawn as dashed lines and, for the sake of clarity, the H atoms not involved in the motif shown have been omitted.

**Figure 7:**
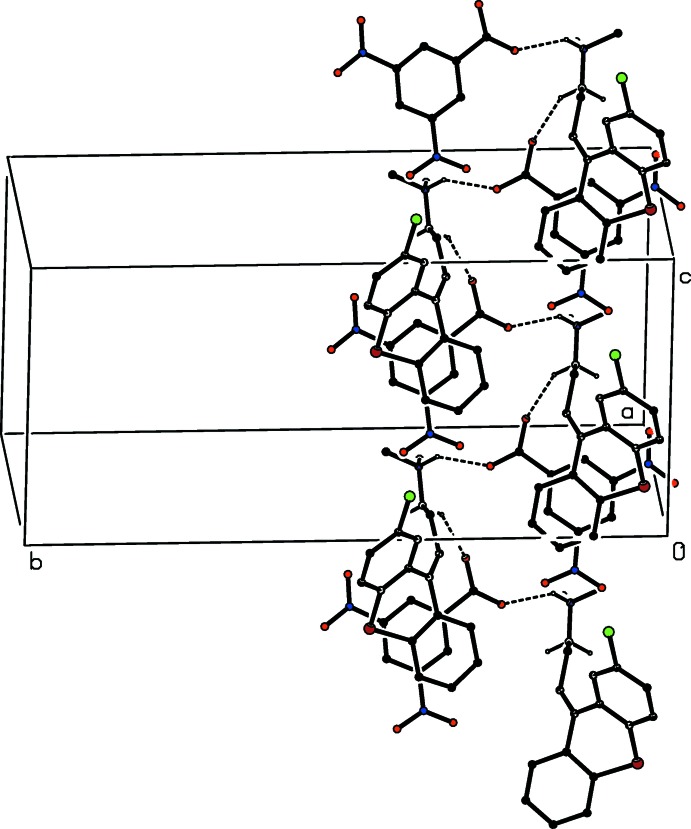
Part of the crystal structure of compound (II) showing the formation of a hydrogen-bonded 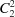
(7) chain running parallel to the [001] direction. Hydrogen bonds are drawn as dashed lines and, for the sake of clarity, the H atoms bonded to the C atoms which are not involved in the motif shown have been omitted.

In the structure of compound (II) there are just two C—H⋯O hydrogen bonds linking the ion-pairs (Fig. 2 ▸, Table 2 ▸) into sheets, whose formation is most easily analysed in terms of two simple-one-dimensional sub-structures (Ferguson *et al.*, 1998*a* ▸,*b* ▸; Gregson *et al.*, 2000 ▸). The hydrogen bond involving atom C14 as the donor links the ions into a 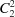
(7) chain running parallel to the [010] direction (Fig. 6 ▸), while that having atom C1 as the donor generates a second 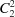
(7) chain, this time running parallel to the [001] direction (Fig. 7 ▸). The combination of chains running parallel to [010] and [001] suffices to generate a sheet lying parallel to (100). The only significant π–π stacking inter­actions lie within the hydrogen-bonded sheets, rather than between adjacent sheets, so that the supra­molecular assembly is strictly two-dimensional.

## Database survey   

It is of inter­est briefly to note the structures of some compounds related to (I) and (II). In neutral trihexyphenidyl, there is an intra­molecular O—H⋯N hydrogen bond forming an *S*(6) motif, but there are no significant direction-specific inter­actions between the mol­ecules (Camerman & Camerman, 1972 ▸), while in the hydro­chloride salt (Maccaroni *et al.*, 2010 ▸), a combination of O—H⋯Cl and N—H⋯Cl hydrogen bonds links the ions into 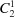
(7) chains. By contrast, in the hydro­chloride salt of procyclidine, which differs from trihexy­phenidyl only in having a pyrrolidine ring in place of the piperidine ring, a combination of O—H⋯Cl and N—H⋯Cl hydrogen bonds generates an 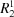
(8) ring, so that the hydrogen-bonded structure consists of ion pairs rather than chains (Camerman & Camerman, 1971 ▸). Neutral 3-(2-chloro-9*H*-thioxanthen-9-yl)-*N*,*N*-di­methyl­propan-1-amine can exist in (*E*) and (*Z*) isomers, and the structures of both forms have been reported (Post *et al.*, 1974 ▸; Sylte & Dahl, 1991 ▸). Flupenthixol (sometimes called flupentixol) is an anti­psychotic agent related to chlorprothixene, but having a tri­fluoro­methyl substituent in place of the chloro substituent and a 4(2-hy­droxy­eth­yl)piperazine substituent in place of the di­methyl­amino group: the structures of both the *E* and *Z* isomers have been reported (Post *et al.*, 1975*a* ▸,*b* ▸), as have those of the di­hydro­chloride salt (Siddegowda *et al.*, 2011 ▸) and the tartrate salt (Yamuna *et al.*, 2014 ▸).

Very short O—H⋯O hydrogen bonds have been reported in a number of acid salts derived from simple carb­oxy­lic acids. In some examples, the anion lies across a symmetry element so that the two O—H distances are identical. Thus, for example, in sodium hydrogendi­acetate the anion lies across a twofold rotation axis with an O⋯O distance of 2.475 (2) Å (Barrow *et al.*, 1975 ▸), while in the analogous potassium salt, which is polymeric, the asymmetric O—H⋯O unit has an O⋯O distance of 2.486 Å [CSD (Groom *et al.*, 2016 ▸) refcode KHACET02; Courtney & Fronczec, 2005 ▸: there are no s.u. values given for the deposited atomic coordinates]. In potassium hydrogenbis(tri­chloro­acetate) (CSD refcode KBTCAC02; Muir *et al.*, 2001 ▸), the asymmetric hydrogen bond has an O⋯O distance of 2.4496 Å (again, there are no s.u. values for the deposited atomic coordinates). By contrast, the anion in sodium hydrogenbis(phen­oxy­acetate) lies across a twofold rotation axis with an O⋯O distance of 2.413 (2) Å (Evans *et al.*, 2001 ▸). The anions in both ethyl­enedi­ammonium hydrogenbis(3,5-di­nitro­benzoate (Jones *et al.*, 2005 ▸) and 2-pyridyl-4′-pyridinium hydrogenbis(3,5-di­nitro­benzoate (Chantrapromma *et al.*, 2002 ▸) lie in a general position, with O⋯O distances of 2.507 (2) and 2.579 (2) Å, respectively, in asymmetric O—H⋯O hydrogen bonds. Finally, we note the extremely short O⋯O distance of 2.29 (2) Å reported for the simple anion [H(OH)_2_]^−^, which lies across a centre of inversion in a mixed salt containing both sodium and methyl­tri­ethyl­ammonium cations, as well as tris­(thio­benzo­hydroximato)chromium(III) anions and water mol­ecules (Abu-Dari *et al.*, 1979 ▸).

## Synthesis and crystallization   

Samples of racemic trihexyphenidine and (Z)-chlorprothixene were gifts from RL Fine Chem Pvt. Ltd., Bengaluru, India. For the synthesis of compound (I), equimolar qu­anti­ties of trihexyphenidine and 3,5-di­nitro­benzoic acid (0.33 mmol of each) were dissolved in hot methanol (10 ml) and the resulting solution was stirred at 333 K for 30 min. The solution was then allowed to cool to ambient temperature, and the resulting crystalline product was collected by filtration. For the synthesis of (II), equimolar qu­anti­ties of chlorprothixene and 3,5-di­nitro­benzoic acid (0.60 mmol of each) were dissolved in hot methanol (10 ml) and the resulting solution was stirred at 333 K for 10 min. The solution was then allowed to cool to ambient temperature, and the resulting crystalline product was collected by filtration. Use of initial molar ratios in the range 5:1 to 1:5 always yielded the same products (I) and (II). Crystals of (I) and (II) suitable for single-crystal X-ray diffraction were grown by slow evaporation, at ambient temperature and in the presence of air, of solutions in methanol–di­methyl­sulfoxide (1:1, *v*/*v*) and *N*,*N*-di­methyl­formamide.

## Refinement   

Crystal data, data collection and structure refinement details are summarized in Table 3 ▸. All H atoms were located in difference maps. The H atoms bonded to C atoms were then treated as riding atoms in geometrically idealized positions with C—H distances 0.95 Å (aromatic), 0.98 Å (CH_3_), 0.99 Å (CH_2_) or 1.00 Å (aliphatic C—H) and with *U*_iso_(H) = *kU*_eq_(C), where *k* = 1.5 for the methyl groups, which were permitted to rotate but not to tilt, and 1.2 for all other H atoms bonded to C atoms. For atom H312 in the short O—H⋯O hydrogen bond, the atomic coordinates and the *U*_iso_(H) value were all refined; for the remaining H atoms bonded to N or O atoms, the atomic coordinates were refined with *U*_iso_(H) = 1.2*U*_eq_(N) or 1.5*U*_eq_(O). The resulting N—H and O—H distances are given in Tables 1 ▸ and 2 ▸. For compound (II), the largest peak in the final difference map, 0.53 e Å^−3^, was located near the bond C17—H17, at distances from these two atoms of 1.40 and 0.62 Å, but no plausible chemical inter­pretation of this seems possible.

## Supplementary Material

Crystal structure: contains datablock(s) global, I, II. DOI: 10.1107/S2056989019001385/zl2748sup1.cif

Structure factors: contains datablock(s) I. DOI: 10.1107/S2056989019001385/zl2748Isup2.hkl

Structure factors: contains datablock(s) II. DOI: 10.1107/S2056989019001385/zl2748IIsup3.hkl

CCDC references: 1883983, 1883984

Additional supporting information:  crystallographic information; 3D view; checkCIF report

## Figures and Tables

**Table 1:** Hydrogen-bond geometry (Å, °) for (I)

*D*—H⋯*A*	*D*—H	H⋯*A*	*D*⋯*A*	*D*—H⋯*A*
O1—H1⋯O412	0.84 (2)	1.91 (2)	2.724 (2)	165.9 (16)
N31—H31⋯O411	0.942 (18)	1.762 (18)	2.7026 (18)	175.7 (14)
C33—H33*A*⋯O452^i^	0.99	2.49	3.4202 (17)	157
C36—H36*A*⋯O1^ii^	0.99	2.56	3.4025 (16)	144
C36—H36*A*⋯O412^ii^	0.99	2.51	3.3949 (19)	148

Symmetry codes: (i) 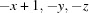; (ii) 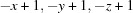.

**Table 2:** Hydrogen-bond geometry (Å, °) for (II)

*D*—H⋯*A*	*D*—H	H⋯*A*	*D*⋯*A*	*D*—H⋯*A*
N1—H1⋯O212	0.866 (17)	2.046 (17)	2.7476 (16)	137.9 (15)
N1—H1⋯O311	0.866 (17)	2.485 (17)	2.9848 (16)	117.5 (14)
O312—H312⋯O211	1.04 (3)	1.41 (3)	2.4197 (15)	161 (3)
C1—H1*B*⋯O211^i^	0.99	2.36	3.2621 (18)	151
C14—H14⋯O232^ii^	0.95	2.41	3.2917 (19)	155
C18—H18⋯O231	0.95	2.53	3.4386 (19)	161

Symmetry codes: (i) 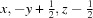; (ii) 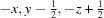.

**Table 3:** Experimental details

	(I)	(II)
Crystal data		
Chemical formula	C_20_H_32_NO^+^·C_7_H_3_N_2_O_6_^−^	C_18_H_19_ClNS^+^·C_7_H_3_N_2_O_6_^−^·C_7_H_4_N_2_O_6_
*M*_r_	513.58	740.09
Crystal system, space group	Triclinic, *P* 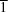	Monoclinic, *P*2_1_/*c*
Temperature (K)	173	173
*a*, *b*, *c* (Å)	11.2743 (12), 11.2898 (12), 12.6478 (13)	11.3454 (8), 24.3857 (16), 11.6098 (8)
α, β, γ (°)	111.923 (1), 114.325 (1), 95.903 (1)	90, 93.691 (1), 90
*V* (Å^3^)	1296.6 (2)	3205.4 (4)
*Z*	2	4
Radiation type	Mo *K*α	Mo *K*α
μ (mm^−1^)	0.10	0.26
Crystal size (mm)	0.61 × 0.58 × 0.13	0.57 × 0.32 × 0.30

Data collection		
Diffractometer	Bruker APEXII CCD	Bruker APEXII CCD
Absorption correction	Multi-scan (*SADABS*; Bruker, 2015 ▸)	Multi-scan (*SADABS*; Bruker, 2015 ▸)
*T*_min_, *T*_max_	0.944, 0.988	0.866, 0.927
No. of measured, independent and observed [*I* > 2σ(*I*)] reflections	7191, 5525, 4819	17539, 7139, 6365
*R*_int_	0.013	0.022
(sin θ/λ)_max_ (Å^−1^)	0.650	0.648

Refinement		
*R*[*F* ^2^ > 2σ(*F* ^2^)], *wR*(*F* ^2^), *S*	0.040, 0.109, 1.07	0.036, 0.098, 1.04
No. of reflections	5525	7139
No. of parameters	340	469
H-atom treatment	H atoms treated by a mixture of independent and constrained refinement	H atoms treated by a mixture of independent and constrained refinement
Δρ_max_, Δρ_min_ (e Å^−3^)	0.32, −0.22	0.53, −0.40

Computer programs: *APEX2* (Bruker, 2014 ▸), *SAINT* (Bruker, 2015 ▸), *SHELXS97* (Sheldrick, 2008 ▸), *SHELXL2014* (Sheldrick, 2015 ▸) and *PLATON* (Spek, 2009 ▸).

## supplementary crystallographic information

### 1-(3-Cyclohexyl-3-hydroxy-3-phenylpropyl)piperidin-1-ium 3,5-dinitrobenzoate (I) Crystal data

**Table:**

C_20_H_32_NO^+^·C_7_H_3_N_2_O_6_^−^	*Z* = 2
*M**_r_* = 513.58	*F*(000) = 548
Triclinic, *P*1	*D*_x_ = 1.315 Mg m^−^^3^
*a* = 11.2743 (12) Å	Mo *K*α radiation, λ = 0.71073 Å
*b* = 11.2898 (12) Å	Cell parameters from 5525 reflections
*c* = 12.6478 (13) Å	θ = 2.0–27.5°
α = 111.923 (1)°	µ = 0.10 mm^−^^1^
β = 114.325 (1)°	*T* = 173 K
γ = 95.903 (1)°	Plate, colourless
*V* = 1296.6 (2) Å^3^	0.61 × 0.58 × 0.13 mm

### 1-(3-Cyclohexyl-3-hydroxy-3-phenylpropyl)piperidin-1-ium 3,5-dinitrobenzoate (I) Data collection

**Table:**

Bruker APEXII CCD diffractometer	5525 independent reflections
Radiation source: fine focus sealed tube	4819 reflections with *I* > 2σ(*I*)
Graphite monochromator	*R*_int_ = 0.013
Detector resolution: 0.3333 pixels mm^-1^	θ_max_ = 27.5°, θ_min_ = 2.0°
φ and ω scans	*h* = −7→14
Absorption correction: multi-scan (SADABS; Bruker, 2015)	*k* = −14→13
*T*_min_ = 0.944, *T*_max_ = 0.988	*l* = −16→15
7191 measured reflections	

### 1-(3-Cyclohexyl-3-hydroxy-3-phenylpropyl)piperidin-1-ium 3,5-dinitrobenzoate (I) Refinement

**Table:**

Refinement on *F*^2^	0 restraints
Least-squares matrix: full	Hydrogen site location: mixed
*R*[*F*^2^ > 2σ(*F*^2^)] = 0.040	H atoms treated by a mixture of independent and constrained refinement
*wR*(*F*^2^) = 0.109	*w* = 1/[σ^2^(*F*_o_^2^) + (0.0513*P*)^2^ + 0.3666*P*] where *P* = (*F*_o_^2^ + 2*F*_c_^2^)/3
*S* = 1.07	(Δ/σ)_max_ < 0.001
5525 reflections	Δρ_max_ = 0.32 e Å^−^^3^
340 parameters	Δρ_min_ = −0.22 e Å^−^^3^

### 1-(3-Cyclohexyl-3-hydroxy-3-phenylpropyl)piperidin-1-ium 3,5-dinitrobenzoate (I) Special details

**Table:**

Geometry. All esds (except the esd in the dihedral angle between two l.s. planes) are estimated using the full covariance matrix. The cell esds are taken into account individually in the estimation of esds in distances, angles and torsion angles; correlations between esds in cell parameters are only used when they are defined by crystal symmetry. An approximate (isotropic) treatment of cell esds is used for estimating esds involving l.s. planes.

### 1-(3-Cyclohexyl-3-hydroxy-3-phenylpropyl)piperidin-1-ium 3,5-dinitrobenzoate (I) Fractional atomic coordinates and isotropic or equivalent isotropic displacement parameters (Å^2^)

**Table:**

	*x*	*y*	*z*	*U*_iso_*/*U*_eq_	
C1	0.12573 (11)	0.36273 (11)	0.26416 (10)	0.0190 (2)	
O1	0.21852 (9)	0.39505 (9)	0.39566 (8)	0.0248 (2)	
H1	0.2717 (18)	0.3492 (17)	0.3928 (16)	0.037*	
C2	0.19972 (12)	0.32806 (11)	0.18280 (11)	0.0208 (2)	
H2A	0.2296	0.2489	0.1838	0.025*	
H2B	0.1354	0.3047	0.0911	0.025*	
C3	0.32358 (12)	0.44489 (12)	0.23607 (12)	0.0225 (2)	
H3A	0.2942	0.5043	0.1959	0.027*	
H3B	0.3615	0.4976	0.3315	0.027*	
C11	0.07862 (11)	0.48561 (11)	0.26615 (11)	0.0199 (2)	
C12	−0.00040 (13)	0.48954 (13)	0.14936 (12)	0.0266 (3)	
H12	−0.0193	0.4184	0.0688	0.032*	
C13	−0.05172 (14)	0.59666 (15)	0.14980 (14)	0.0329 (3)	
H13	−0.1029	0.5994	0.0699	0.039*	
C14	−0.02874 (15)	0.69892 (14)	0.26556 (15)	0.0343 (3)	
H14	−0.0662	0.7705	0.2652	0.041*	
C15	0.04917 (14)	0.69630 (13)	0.38178 (14)	0.0312 (3)	
H15	0.0659	0.7667	0.4618	0.037*	
C16	0.10342 (13)	0.59084 (12)	0.38237 (12)	0.0262 (3)	
H16	0.1580	0.5908	0.4630	0.031*	
C21	−0.00168 (12)	0.24305 (11)	0.20637 (11)	0.0213 (2)	
H21	−0.0682	0.2300	0.1179	0.026*	
C22	0.03208 (14)	0.11178 (13)	0.19039 (14)	0.0304 (3)	
H22A	0.1027	0.1243	0.2761	0.037*	
H22B	0.0703	0.0885	0.1301	0.037*	
C23	−0.09347 (16)	−0.00389 (13)	0.13718 (15)	0.0358 (3)	
H23A	−0.1593	−0.0241	0.0468	0.043*	
H23B	−0.0654	−0.0848	0.1343	0.043*	
C24	−0.16323 (16)	0.02887 (14)	0.22054 (15)	0.0373 (3)	
H24A	−0.2476	−0.0453	0.1795	0.045*	
H24B	−0.1018	0.0384	0.3080	0.045*	
C25	−0.19849 (15)	0.15810 (14)	0.23433 (16)	0.0373 (3)	
H25A	−0.2393	0.1807	0.2925	0.045*	
H25B	−0.2670	0.1454	0.1477	0.045*	
C26	−0.07169 (14)	0.27333 (13)	0.29049 (14)	0.0295 (3)	
H26A	−0.0063	0.2902	0.3798	0.035*	
H26B	−0.0982	0.3556	0.2967	0.035*	
N31	0.43308 (10)	0.39959 (10)	0.20951 (9)	0.0199 (2)	
H31	0.4499 (14)	0.3329 (15)	0.2365 (14)	0.024*	
C32	0.39085 (13)	0.33636 (15)	0.06744 (12)	0.0297 (3)	
H32A	0.3700	0.4022	0.0337	0.036*	
H32B	0.3070	0.2594	0.0189	0.036*	
C33	0.50410 (14)	0.28851 (17)	0.04548 (13)	0.0387 (3)	
H33A	0.4756	0.2497	−0.0487	0.046*	
H33B	0.5196	0.2174	0.0728	0.046*	
C34	0.63655 (15)	0.40357 (17)	0.12266 (15)	0.0400 (3)	
H34A	0.7101	0.3692	0.1114	0.048*	
H34B	0.6241	0.4705	0.0893	0.048*	
C35	0.67691 (14)	0.46939 (15)	0.26640 (14)	0.0349 (3)	
H35A	0.6995	0.4051	0.3019	0.042*	
H35B	0.7594	0.5478	0.3149	0.042*	
C36	0.56213 (13)	0.51417 (13)	0.28592 (13)	0.0314 (3)	
H36A	0.5894	0.5532	0.3798	0.038*	
H36B	0.5455	0.5847	0.2580	0.038*	
C41	0.57048 (12)	0.14707 (12)	0.45254 (12)	0.0236 (2)	
C42	0.60720 (13)	0.19029 (12)	0.58292 (12)	0.0251 (3)	
H42	0.5743	0.2571	0.6242	0.030*	
C43	0.69222 (13)	0.13517 (12)	0.65226 (12)	0.0260 (3)	
C44	0.73907 (13)	0.03474 (13)	0.59588 (13)	0.0273 (3)	
H44	0.7981	−0.0018	0.6448	0.033*	
C45	0.69577 (13)	−0.00984 (12)	0.46483 (12)	0.0246 (3)	
C46	0.61418 (12)	0.04496 (12)	0.39155 (12)	0.0231 (2)	
H46	0.5889	0.0134	0.3020	0.028*	
C411	0.48388 (14)	0.21494 (13)	0.37987 (13)	0.0298 (3)	
O411	0.48586 (11)	0.20351 (11)	0.27857 (10)	0.0394 (3)	
O412	0.42093 (13)	0.27683 (14)	0.42999 (11)	0.0571 (4)	
N43	0.73412 (14)	0.18513 (13)	0.79160 (12)	0.0387 (3)	
O431	0.68879 (14)	0.27080 (13)	0.83989 (11)	0.0555 (3)	
O432	0.81408 (17)	0.13945 (15)	0.85243 (12)	0.0690 (4)	
N45	0.73858 (13)	−0.12129 (12)	0.40094 (12)	0.0341 (3)	
O451	0.83761 (16)	−0.14320 (16)	0.46933 (13)	0.0756 (5)	
O452	0.67240 (12)	−0.18761 (10)	0.28288 (10)	0.0433 (3)	

### 1-(3-Cyclohexyl-3-hydroxy-3-phenylpropyl)piperidin-1-ium 3,5-dinitrobenzoate (I) Atomic displacement parameters (Å^2^)

**Table:**

	*U*^11^	*U*^22^	*U*^33^	*U*^12^	*U*^13^	*U*^23^
C1	0.0193 (5)	0.0205 (5)	0.0185 (5)	0.0088 (4)	0.0076 (4)	0.0113 (4)
O1	0.0246 (4)	0.0304 (5)	0.0200 (4)	0.0149 (4)	0.0079 (3)	0.0138 (3)
C2	0.0215 (6)	0.0198 (5)	0.0226 (5)	0.0083 (4)	0.0106 (4)	0.0109 (4)
C3	0.0228 (6)	0.0197 (5)	0.0284 (6)	0.0097 (5)	0.0135 (5)	0.0122 (5)
C11	0.0186 (5)	0.0205 (5)	0.0254 (6)	0.0078 (4)	0.0111 (5)	0.0141 (4)
C12	0.0270 (6)	0.0309 (6)	0.0272 (6)	0.0139 (5)	0.0129 (5)	0.0177 (5)
C13	0.0325 (7)	0.0411 (8)	0.0423 (7)	0.0212 (6)	0.0195 (6)	0.0317 (6)
C14	0.0382 (8)	0.0309 (7)	0.0569 (9)	0.0223 (6)	0.0311 (7)	0.0305 (7)
C15	0.0370 (7)	0.0220 (6)	0.0412 (7)	0.0119 (5)	0.0243 (6)	0.0141 (5)
C16	0.0285 (6)	0.0233 (6)	0.0277 (6)	0.0098 (5)	0.0133 (5)	0.0126 (5)
C21	0.0220 (6)	0.0211 (6)	0.0241 (5)	0.0081 (5)	0.0109 (5)	0.0134 (4)
C22	0.0348 (7)	0.0226 (6)	0.0418 (7)	0.0121 (5)	0.0234 (6)	0.0163 (5)
C23	0.0438 (8)	0.0223 (6)	0.0441 (8)	0.0077 (6)	0.0258 (7)	0.0136 (6)
C24	0.0436 (8)	0.0274 (7)	0.0463 (8)	0.0052 (6)	0.0271 (7)	0.0179 (6)
C25	0.0354 (8)	0.0332 (7)	0.0540 (9)	0.0105 (6)	0.0295 (7)	0.0216 (6)
C26	0.0325 (7)	0.0246 (6)	0.0402 (7)	0.0108 (5)	0.0238 (6)	0.0159 (5)
N31	0.0189 (5)	0.0217 (5)	0.0220 (5)	0.0080 (4)	0.0092 (4)	0.0132 (4)
C32	0.0229 (6)	0.0426 (7)	0.0208 (6)	0.0083 (5)	0.0083 (5)	0.0148 (5)
C33	0.0294 (7)	0.0573 (9)	0.0252 (6)	0.0126 (7)	0.0154 (6)	0.0127 (6)
C34	0.0297 (7)	0.0611 (10)	0.0410 (8)	0.0148 (7)	0.0222 (6)	0.0290 (7)
C35	0.0204 (6)	0.0411 (8)	0.0362 (7)	0.0053 (6)	0.0116 (5)	0.0146 (6)
C36	0.0225 (6)	0.0275 (6)	0.0341 (7)	0.0025 (5)	0.0112 (5)	0.0087 (5)
C41	0.0199 (6)	0.0206 (5)	0.0314 (6)	0.0074 (5)	0.0102 (5)	0.0154 (5)
C42	0.0256 (6)	0.0197 (6)	0.0322 (6)	0.0090 (5)	0.0146 (5)	0.0131 (5)
C43	0.0297 (6)	0.0236 (6)	0.0273 (6)	0.0083 (5)	0.0137 (5)	0.0144 (5)
C44	0.0305 (7)	0.0274 (6)	0.0321 (6)	0.0144 (5)	0.0148 (5)	0.0205 (5)
C45	0.0264 (6)	0.0221 (6)	0.0329 (6)	0.0120 (5)	0.0162 (5)	0.0169 (5)
C46	0.0228 (6)	0.0215 (6)	0.0279 (6)	0.0077 (5)	0.0116 (5)	0.0149 (5)
C411	0.0291 (7)	0.0258 (6)	0.0328 (7)	0.0148 (5)	0.0100 (5)	0.0158 (5)
O411	0.0495 (6)	0.0435 (6)	0.0468 (6)	0.0300 (5)	0.0264 (5)	0.0343 (5)
O412	0.0659 (8)	0.0780 (9)	0.0382 (6)	0.0600 (7)	0.0233 (6)	0.0298 (6)
N43	0.0522 (8)	0.0376 (7)	0.0314 (6)	0.0204 (6)	0.0202 (6)	0.0192 (5)
O431	0.0820 (9)	0.0577 (7)	0.0393 (6)	0.0412 (7)	0.0359 (6)	0.0222 (5)
O432	0.1064 (11)	0.0804 (9)	0.0373 (6)	0.0633 (9)	0.0315 (7)	0.0383 (6)
N45	0.0459 (7)	0.0346 (6)	0.0365 (6)	0.0258 (6)	0.0237 (6)	0.0231 (5)
O451	0.0945 (11)	0.1011 (11)	0.0485 (7)	0.0853 (10)	0.0330 (7)	0.0385 (7)
O452	0.0612 (7)	0.0328 (5)	0.0372 (6)	0.0235 (5)	0.0239 (5)	0.0151 (4)

### 1-(3-Cyclohexyl-3-hydroxy-3-phenylpropyl)piperidin-1-ium 3,5-dinitrobenzoate (I) Geometric parameters (Å, º)

**Table:**

C1—O1	1.4243 (13)	C26—H26A	0.9900
C1—C11	1.5310 (15)	C26—H26B	0.9900
C1—C2	1.5432 (16)	N31—C32	1.4941 (15)
C1—C21	1.5562 (16)	N31—C36	1.4984 (15)
O1—H1	0.834 (18)	N31—H31	0.942 (15)
C2—C3	1.5252 (16)	C32—C33	1.5236 (19)
C2—H2A	0.9900	C32—H32A	0.9900
C2—H2B	0.9900	C32—H32B	0.9900
C3—N31	1.4972 (15)	C33—C34	1.524 (2)
C3—H3A	0.9900	C33—H33A	0.9900
C3—H3B	0.9900	C33—H33B	0.9900
C11—C16	1.3926 (17)	C34—C35	1.518 (2)
C11—C12	1.3966 (16)	C34—H34A	0.9900
C12—C13	1.3924 (18)	C34—H34B	0.9900
C12—H12	0.9500	C35—C36	1.5151 (19)
C13—C14	1.381 (2)	C35—H35A	0.9900
C13—H13	0.9500	C35—H35B	0.9900
C14—C15	1.381 (2)	C36—H36A	0.9900
C14—H14	0.9500	C36—H36B	0.9900
C15—C16	1.3945 (18)	C41—C42	1.3870 (18)
C15—H15	0.9500	C41—C46	1.3902 (17)
C16—H16	0.9500	C41—C411	1.5250 (16)
C21—C22	1.5290 (16)	C42—C43	1.3831 (17)
C21—C26	1.5322 (17)	C42—H42	0.9500
C21—H21	1.0000	C43—C44	1.3794 (18)
C22—C23	1.5298 (19)	C43—N43	1.4712 (17)
C22—H22A	0.9900	C44—C45	1.3780 (18)
C22—H22B	0.9900	C44—H44	0.9500
C23—C24	1.523 (2)	C45—C46	1.3895 (16)
C23—H23A	0.9900	C45—N45	1.4684 (16)
C23—H23B	0.9900	C46—H46	0.9500
C24—C25	1.518 (2)	C411—O412	1.2392 (17)
C24—H24A	0.9900	C411—O411	1.2488 (17)
C24—H24B	0.9900	N43—O431	1.2195 (17)
C25—C26	1.5269 (18)	N43—O432	1.2211 (16)
C25—H25A	0.9900	N45—O451	1.2153 (16)
C25—H25B	0.9900	N45—O452	1.2163 (16)
			
O1—C1—C11	107.31 (9)	C25—C26—H26A	109.2
O1—C1—C2	109.01 (9)	C21—C26—H26A	109.2
C11—C1—C2	111.07 (9)	C25—C26—H26B	109.2
O1—C1—C21	110.45 (9)	C21—C26—H26B	109.2
C11—C1—C21	108.41 (9)	H26A—C26—H26B	107.9
C2—C1—C21	110.55 (9)	C32—N31—C3	112.66 (9)
C1—O1—H1	106.8 (11)	C32—N31—C36	110.68 (10)
C3—C2—C1	111.54 (9)	C3—N31—C36	110.31 (9)
C3—C2—H2A	109.3	C32—N31—H31	106.4 (9)
C1—C2—H2A	109.3	C3—N31—H31	107.9 (9)
C3—C2—H2B	109.3	C36—N31—H31	108.8 (9)
C1—C2—H2B	109.3	N31—C32—C33	110.53 (10)
H2A—C2—H2B	108.0	N31—C32—H32A	109.5
N31—C3—C2	112.50 (9)	C33—C32—H32A	109.5
N31—C3—H3A	109.1	N31—C32—H32B	109.5
C2—C3—H3A	109.1	C33—C32—H32B	109.5
N31—C3—H3B	109.1	H32A—C32—H32B	108.1
C2—C3—H3B	109.1	C32—C33—C34	111.08 (13)
H3A—C3—H3B	107.8	C32—C33—H33A	109.4
C16—C11—C12	118.22 (11)	C34—C33—H33A	109.4
C16—C11—C1	121.30 (10)	C32—C33—H33B	109.4
C12—C11—C1	120.27 (11)	C34—C33—H33B	109.4
C13—C12—C11	120.64 (12)	H33A—C33—H33B	108.0
C13—C12—H12	119.7	C35—C34—C33	109.98 (11)
C11—C12—H12	119.7	C35—C34—H34A	109.7
C14—C13—C12	120.50 (12)	C33—C34—H34A	109.7
C14—C13—H13	119.8	C35—C34—H34B	109.7
C12—C13—H13	119.8	C33—C34—H34B	109.7
C13—C14—C15	119.46 (12)	H34A—C34—H34B	108.2
C13—C14—H14	120.3	C36—C35—C34	110.94 (11)
C15—C14—H14	120.3	C36—C35—H35A	109.5
C14—C15—C16	120.36 (13)	C34—C35—H35A	109.5
C14—C15—H15	119.8	C36—C35—H35B	109.5
C16—C15—H15	119.8	C34—C35—H35B	109.5
C11—C16—C15	120.79 (12)	H35A—C35—H35B	108.0
C11—C16—H16	119.6	N31—C36—C35	111.39 (11)
C15—C16—H16	119.6	N31—C36—H36A	109.3
C22—C21—C26	109.28 (10)	C35—C36—H36A	109.3
C22—C21—C1	112.90 (10)	N31—C36—H36B	109.3
C26—C21—C1	111.07 (10)	C35—C36—H36B	109.3
C22—C21—H21	107.8	H36A—C36—H36B	108.0
C26—C21—H21	107.8	C42—C41—C46	119.83 (11)
C1—C21—H21	107.8	C42—C41—C411	118.64 (11)
C21—C22—C23	112.23 (11)	C46—C41—C411	121.53 (11)
C21—C22—H22A	109.2	C43—C42—C41	119.35 (11)
C23—C22—H22A	109.2	C43—C42—H42	120.3
C21—C22—H22B	109.2	C41—C42—H42	120.3
C23—C22—H22B	109.2	C44—C43—C42	122.62 (12)
H22A—C22—H22B	107.9	C44—C43—N43	118.58 (11)
C24—C23—C22	112.00 (12)	C42—C43—N43	118.80 (12)
C24—C23—H23A	109.2	C45—C44—C43	116.45 (11)
C22—C23—H23A	109.2	C45—C44—H44	121.8
C24—C23—H23B	109.2	C43—C44—H44	121.8
C22—C23—H23B	109.2	C44—C45—C46	123.31 (12)
H23A—C23—H23B	107.9	C44—C45—N45	117.53 (11)
C25—C24—C23	110.25 (12)	C46—C45—N45	119.15 (11)
C25—C24—H24A	109.6	C45—C46—C41	118.33 (11)
C23—C24—H24A	109.6	C45—C46—H46	120.8
C25—C24—H24B	109.6	C41—C46—H46	120.8
C23—C24—H24B	109.6	O412—C411—O411	127.63 (12)
H24A—C24—H24B	108.1	O412—C411—C41	115.60 (12)
C24—C25—C26	111.10 (12)	O411—C411—C41	116.77 (11)
C24—C25—H25A	109.4	O431—N43—O432	123.94 (13)
C26—C25—H25A	109.4	O431—N43—C43	118.29 (12)
C24—C25—H25B	109.4	O432—N43—C43	117.76 (12)
C26—C25—H25B	109.4	O451—N45—O452	123.51 (13)
H25A—C25—H25B	108.0	O451—N45—C45	118.05 (12)
C25—C26—C21	111.98 (11)	O452—N45—C45	118.43 (11)
			
O1—C1—C2—C3	−59.48 (12)	C2—C3—N31—C36	−168.65 (10)
C11—C1—C2—C3	58.55 (12)	C3—N31—C32—C33	−178.36 (11)
C21—C1—C2—C3	178.93 (9)	C36—N31—C32—C33	57.61 (14)
C1—C2—C3—N31	151.06 (9)	N31—C32—C33—C34	−57.20 (16)
O1—C1—C11—C16	−13.05 (15)	C32—C33—C34—C35	55.72 (17)
C2—C1—C11—C16	−132.11 (11)	C33—C34—C35—C36	−55.20 (17)
C21—C1—C11—C16	106.24 (12)	C32—N31—C36—C35	−57.67 (14)
O1—C1—C11—C12	172.21 (10)	C3—N31—C36—C35	176.97 (10)
C2—C1—C11—C12	53.16 (14)	C34—C35—C36—N31	56.62 (16)
C21—C1—C11—C12	−68.49 (13)	C46—C41—C42—C43	2.93 (18)
C16—C11—C12—C13	0.57 (19)	C411—C41—C42—C43	−176.29 (11)
C1—C11—C12—C13	175.46 (12)	C41—C42—C43—C44	−2.27 (19)
C11—C12—C13—C14	−2.0 (2)	C41—C42—C43—N43	177.95 (11)
C12—C13—C14—C15	1.9 (2)	C42—C43—C44—C45	−0.52 (19)
C13—C14—C15—C16	−0.5 (2)	N43—C43—C44—C45	179.25 (12)
C12—C11—C16—C15	0.85 (18)	C43—C44—C45—C46	2.75 (19)
C1—C11—C16—C15	−173.99 (11)	C43—C44—C45—N45	−177.01 (11)
C14—C15—C16—C11	−0.9 (2)	C44—C45—C46—C41	−2.10 (19)
O1—C1—C21—C22	−66.93 (13)	N45—C45—C46—C41	177.65 (11)
C11—C1—C21—C22	175.76 (10)	C42—C41—C46—C45	−0.84 (18)
C2—C1—C21—C22	53.80 (12)	C411—C41—C46—C45	178.36 (11)
O1—C1—C21—C26	56.22 (12)	C42—C41—C411—O412	−19.47 (18)
C11—C1—C21—C26	−61.08 (12)	C46—C41—C411—O412	161.32 (13)
C2—C1—C21—C26	176.96 (9)	C42—C41—C411—O411	159.59 (12)
C26—C21—C22—C23	54.22 (15)	C46—C41—C411—O411	−19.62 (18)
C1—C21—C22—C23	178.36 (10)	C44—C43—N43—O431	−177.62 (14)
C21—C22—C23—C24	−55.07 (16)	C42—C43—N43—O431	2.2 (2)
C22—C23—C24—C25	54.99 (17)	C44—C43—N43—O432	3.4 (2)
C23—C24—C25—C26	−56.14 (17)	C42—C43—N43—O432	−176.77 (14)
C24—C25—C26—C21	57.78 (15)	C44—C45—N45—O451	−20.5 (2)
C22—C21—C26—C25	−55.75 (14)	C46—C45—N45—O451	159.71 (14)
C1—C21—C26—C25	179.04 (10)	C44—C45—N45—O452	158.66 (13)
C2—C3—N31—C32	67.11 (12)	C46—C45—N45—O452	−21.11 (18)

### 1-(3-Cyclohexyl-3-hydroxy-3-phenylpropyl)piperidin-1-ium 3,5-dinitrobenzoate (I) Hydrogen-bond geometry (Å, º)

**Table:**

*D*—H···*A*	*D*—H	H···*A*	*D*···*A*	*D*—H···*A*
O1—H1···O412	0.84 (2)	1.91 (2)	2.724 (2)	165.9 (16)
N31—H31···O411	0.942 (18)	1.762 (18)	2.7026 (18)	175.7 (14)
C33—H33*A*···O452^i^	0.99	2.49	3.4202 (17)	157
C36—H36*A*···O1^ii^	0.99	2.56	3.4025 (16)	144
C36—H36*A*···O412^ii^	0.99	2.51	3.3949 (19)	148

Symmetry codes: (i) −*x*+1, −*y*, −*z*; (ii) −*x*+1, −*y*+1, −*z*+1.

### (Z)-3-(2-Chloro-9H-thioxanthen-9-yl)-N,N-dimethylpropan-1-aminium hydrogen bis(3,5-dinitrobenzoate) (II) Crystal data

**Table:**

C_18_H_19_ClNS^+^·C_7_H_3_N_2_O_6_^−^·C_7_H_4_N_2_O_6_	*F*(000) = 1528
*M**_r_* = 740.09	*D*_x_ = 1.534 Mg m^−^^3^
Monoclinic, *P*2_1_/*c*	Mo *K*α radiation, λ = 0.71073 Å
*a* = 11.3454 (8) Å	Cell parameters from 7139 reflections
*b* = 24.3857 (16) Å	θ = 1.8–27.4°
*c* = 11.6098 (8) Å	µ = 0.26 mm^−^^1^
β = 93.691 (1)°	*T* = 173 K
*V* = 3205.4 (4) Å^3^	Block, colourless
*Z* = 4	0.57 × 0.32 × 0.30 mm

### (Z)-3-(2-Chloro-9H-thioxanthen-9-yl)-N,N-dimethylpropan-1-aminium hydrogen bis(3,5-dinitrobenzoate) (II) Data collection

**Table:**

Bruker APEXII CCD diffractometer	7139 independent reflections
Radiation source: fine focus sealed tube	6365 reflections with *I* > 2σ(*I*)
Graphite monochromator	*R*_int_ = 0.022
Detector resolution: 0.3333 pixels mm^-1^	θ_max_ = 27.4°, θ_min_ = 1.8°
φ and ω scans	*h* = −14→10
Absorption correction: multi-scan (SADABS; Bruker, 2015)	*k* = −31→29
*T*_min_ = 0.866, *T*_max_ = 0.927	*l* = −14→14
17539 measured reflections	

### (Z)-3-(2-Chloro-9H-thioxanthen-9-yl)-N,N-dimethylpropan-1-aminium hydrogen bis(3,5-dinitrobenzoate) (II) Refinement

**Table:**

Refinement on *F*^2^	0 restraints
Least-squares matrix: full	Hydrogen site location: mixed
*R*[*F*^2^ > 2σ(*F*^2^)] = 0.036	H atoms treated by a mixture of independent and constrained refinement
*wR*(*F*^2^) = 0.098	*w* = 1/[σ^2^(*F*_o_^2^) + (0.0482*P*)^2^ + 1.3713*P*] where *P* = (*F*_o_^2^ + 2*F*_c_^2^)/3
*S* = 1.04	(Δ/σ)_max_ = 0.001
7139 reflections	Δρ_max_ = 0.53 e Å^−^^3^
469 parameters	Δρ_min_ = −0.40 e Å^−^^3^

### (Z)-3-(2-Chloro-9H-thioxanthen-9-yl)-N,N-dimethylpropan-1-aminium hydrogen bis(3,5-dinitrobenzoate) (II) Special details

**Table:**

Geometry. All esds (except the esd in the dihedral angle between two l.s. planes) are estimated using the full covariance matrix. The cell esds are taken into account individually in the estimation of esds in distances, angles and torsion angles; correlations between esds in cell parameters are only used when they are defined by crystal symmetry. An approximate (isotropic) treatment of cell esds is used for estimating esds involving l.s. planes.

### (Z)-3-(2-Chloro-9H-thioxanthen-9-yl)-N,N-dimethylpropan-1-aminium hydrogen bis(3,5-dinitrobenzoate) (II) Fractional atomic coordinates and isotropic or equivalent isotropic displacement parameters (Å^2^)

**Table:**

	*x*	*y*	*z*	*U*_iso_*/*U*_eq_	
N1	0.46269 (10)	0.12939 (5)	0.57731 (10)	0.0223 (2)	
H1	0.4447 (15)	0.1572 (7)	0.6191 (15)	0.027*	
C1	0.38593 (12)	0.13344 (6)	0.46761 (12)	0.0246 (3)	
H1A	0.3964	0.0999	0.4214	0.029*	
H1B	0.4126	0.1650	0.4223	0.029*	
C2	0.25502 (12)	0.14048 (6)	0.48634 (12)	0.0261 (3)	
H2A	0.2230	0.1063	0.5182	0.031*	
H2B	0.2439	0.1706	0.5418	0.031*	
C3	0.19115 (12)	0.15364 (6)	0.37157 (12)	0.0245 (3)	
H4	0.2297	0.1786	0.3237	0.029*	
C11	−0.01624 (12)	0.11072 (5)	0.50768 (12)	0.0239 (3)	
H11	0.0195	0.1416	0.5457	0.029*	
C12	−0.09218 (12)	0.07758 (6)	0.56502 (12)	0.0262 (3)	
Cl12	−0.12220 (4)	0.09270 (2)	0.70590 (3)	0.03840 (11)	
C13	−0.14546 (13)	0.03194 (6)	0.51215 (14)	0.0295 (3)	
H13	−0.1956	0.0089	0.5533	0.035*	
C14	−0.12428 (13)	0.02074 (6)	0.39893 (13)	0.0291 (3)	
H14	−0.1610	−0.0100	0.3615	0.035*	
C14A	−0.04963 (12)	0.05410 (5)	0.33893 (12)	0.0242 (3)	
C14B	−0.01152 (12)	0.10620 (6)	0.13901 (12)	0.0244 (3)	
C15	−0.04959 (12)	0.11668 (7)	0.02414 (12)	0.0290 (3)	
H15	−0.0848	0.0883	−0.0223	0.035*	
C16	−0.03588 (13)	0.16844 (7)	−0.02156 (13)	0.0319 (3)	
H16	−0.0612	0.1756	−0.0997	0.038*	
C17	0.01496 (13)	0.21007 (7)	0.04673 (13)	0.0319 (3)	
H17	0.0233	0.2458	0.0155	0.038*	
C18	0.05355 (12)	0.19964 (6)	0.16036 (12)	0.0275 (3)	
H18	0.0880	0.2284	0.2063	0.033*	
C18A	0.04245 (11)	0.14727 (6)	0.20842 (11)	0.0228 (3)	
C18B	0.00798 (11)	0.09882 (5)	0.39387 (12)	0.0218 (3)	
C19	0.08673 (12)	0.13438 (5)	0.32901 (11)	0.0224 (3)	
S10	−0.02859 (4)	0.03901 (2)	0.19275 (3)	0.03131 (10)	
C111	0.58939 (13)	0.13566 (7)	0.55300 (14)	0.0342 (3)	
H11A	0.6382	0.1352	0.6258	0.051*	
H11B	0.6005	0.1705	0.5132	0.051*	
H11C	0.6128	0.1053	0.5040	0.051*	
C112	0.44364 (15)	0.07721 (6)	0.63983 (13)	0.0339 (3)	
H11D	0.4597	0.0461	0.5897	0.051*	
H11E	0.3617	0.0754	0.6615	0.051*	
H11F	0.4971	0.0757	0.7095	0.051*	
C21	0.34839 (11)	0.33156 (5)	0.58695 (11)	0.0213 (3)	
C22	0.29675 (12)	0.32558 (6)	0.47617 (12)	0.0235 (3)	
H22	0.2962	0.2910	0.4383	0.028*	
C23	0.24587 (12)	0.37130 (6)	0.42191 (12)	0.0246 (3)	
C24	0.24301 (12)	0.42245 (6)	0.47285 (12)	0.0258 (3)	
H24	0.2080	0.4532	0.4337	0.031*	
C25	0.29399 (12)	0.42643 (6)	0.58389 (12)	0.0239 (3)	
C26	0.34720 (11)	0.38231 (6)	0.64140 (12)	0.0226 (3)	
H26	0.3825	0.3867	0.7173	0.027*	
C211	0.40906 (11)	0.28301 (5)	0.64702 (11)	0.0222 (3)	
O211	0.45673 (10)	0.29421 (4)	0.74604 (9)	0.0345 (3)	
O212	0.40959 (10)	0.23880 (4)	0.59644 (9)	0.0335 (2)	
N23	0.19402 (11)	0.36503 (6)	0.30332 (11)	0.0324 (3)	
O231	0.18250 (13)	0.31902 (5)	0.26351 (10)	0.0515 (3)	
O232	0.16581 (13)	0.40671 (5)	0.24991 (11)	0.0484 (3)	
N25	0.29378 (11)	0.48001 (5)	0.64322 (12)	0.0314 (3)	
O251	0.25334 (12)	0.51946 (5)	0.58850 (11)	0.0446 (3)	
O252	0.33426 (12)	0.48210 (5)	0.74342 (11)	0.0454 (3)	
C31	0.63540 (11)	0.17006 (6)	1.00227 (12)	0.0231 (3)	
C32	0.70062 (12)	0.20569 (5)	1.07526 (12)	0.0235 (3)	
H32	0.7188	0.2417	1.0504	0.028*	
C33	0.73852 (12)	0.18786 (6)	1.18466 (12)	0.0251 (3)	
C34	0.71344 (12)	0.13625 (6)	1.22519 (13)	0.0277 (3)	
H34	0.7381	0.1250	1.3013	0.033*	
C35	0.65054 (12)	0.10178 (6)	1.14944 (13)	0.0274 (3)	
C36	0.61247 (12)	0.11696 (6)	1.03830 (13)	0.0255 (3)	
H36	0.5716	0.0917	0.9878	0.031*	
C311	0.57941 (12)	0.18953 (6)	0.88847 (12)	0.0248 (3)	
O311	0.52336 (11)	0.15826 (5)	0.82386 (9)	0.0390 (3)	
O312	0.59247 (10)	0.24171 (4)	0.87186 (9)	0.0317 (2)	
H312	0.533 (3)	0.2576 (13)	0.808 (3)	0.099 (10)*	
N33	0.80516 (11)	0.22598 (5)	1.26284 (11)	0.0321 (3)	
O331	0.82007 (12)	0.27274 (5)	1.22899 (11)	0.0449 (3)	
O332	0.84145 (11)	0.20876 (5)	1.35770 (10)	0.0441 (3)	
N35	0.61594 (12)	0.04780 (5)	1.19234 (13)	0.0363 (3)	
O351	0.62170 (13)	0.04108 (6)	1.29795 (12)	0.0563 (4)	
O352	0.57984 (13)	0.01343 (5)	1.12204 (13)	0.0514 (3)	

### (Z)-3-(2-Chloro-9H-thioxanthen-9-yl)-N,N-dimethylpropan-1-aminium hydrogen bis(3,5-dinitrobenzoate) (II) Atomic displacement parameters (Å^2^)

**Table:**

	*U*^11^	*U*^22^	*U*^33^	*U*^12^	*U*^13^	*U*^23^
N1	0.0241 (6)	0.0184 (5)	0.0237 (6)	−0.0004 (4)	−0.0040 (4)	0.0005 (4)
C1	0.0263 (7)	0.0260 (7)	0.0207 (6)	−0.0005 (5)	−0.0043 (5)	0.0021 (5)
C2	0.0249 (7)	0.0294 (7)	0.0233 (7)	−0.0052 (5)	−0.0043 (5)	0.0028 (5)
C3	0.0239 (6)	0.0254 (7)	0.0239 (7)	−0.0021 (5)	−0.0022 (5)	0.0027 (5)
C11	0.0239 (7)	0.0202 (6)	0.0269 (7)	0.0015 (5)	−0.0040 (5)	0.0014 (5)
C12	0.0251 (7)	0.0259 (7)	0.0273 (7)	0.0052 (5)	−0.0003 (5)	0.0042 (5)
Cl12	0.0427 (2)	0.0438 (2)	0.02942 (19)	0.00309 (17)	0.00775 (15)	0.00411 (15)
C13	0.0239 (7)	0.0250 (7)	0.0393 (8)	−0.0008 (5)	−0.0004 (6)	0.0081 (6)
C14	0.0267 (7)	0.0198 (7)	0.0397 (8)	−0.0019 (5)	−0.0071 (6)	0.0015 (6)
C14A	0.0239 (7)	0.0198 (6)	0.0279 (7)	0.0036 (5)	−0.0063 (5)	0.0000 (5)
C14B	0.0202 (6)	0.0270 (7)	0.0259 (7)	0.0046 (5)	−0.0005 (5)	−0.0018 (5)
C15	0.0236 (7)	0.0390 (8)	0.0240 (7)	0.0046 (6)	−0.0023 (5)	−0.0047 (6)
C16	0.0266 (7)	0.0461 (9)	0.0226 (7)	0.0071 (6)	−0.0014 (5)	0.0033 (6)
C17	0.0279 (7)	0.0369 (8)	0.0308 (8)	0.0029 (6)	0.0015 (6)	0.0100 (6)
C18	0.0230 (7)	0.0296 (7)	0.0297 (7)	−0.0009 (5)	−0.0010 (5)	0.0023 (6)
C18A	0.0175 (6)	0.0275 (7)	0.0233 (6)	0.0022 (5)	−0.0009 (5)	0.0003 (5)
C18B	0.0199 (6)	0.0184 (6)	0.0263 (7)	0.0019 (5)	−0.0045 (5)	0.0017 (5)
C19	0.0225 (6)	0.0206 (6)	0.0235 (6)	0.0017 (5)	−0.0023 (5)	−0.0006 (5)
S10	0.0409 (2)	0.02266 (18)	0.02939 (19)	0.00226 (14)	−0.00557 (15)	−0.00520 (13)
C111	0.0230 (7)	0.0406 (9)	0.0384 (8)	0.0024 (6)	−0.0031 (6)	0.0023 (7)
C112	0.0449 (9)	0.0243 (7)	0.0311 (8)	−0.0040 (6)	−0.0090 (6)	0.0084 (6)
C21	0.0179 (6)	0.0223 (6)	0.0240 (6)	−0.0001 (5)	0.0026 (5)	0.0033 (5)
C22	0.0222 (6)	0.0236 (7)	0.0246 (6)	−0.0027 (5)	0.0014 (5)	0.0018 (5)
C23	0.0209 (6)	0.0296 (7)	0.0230 (7)	−0.0028 (5)	−0.0005 (5)	0.0051 (5)
C24	0.0213 (6)	0.0258 (7)	0.0304 (7)	0.0023 (5)	0.0030 (5)	0.0089 (5)
C25	0.0206 (6)	0.0212 (6)	0.0304 (7)	0.0012 (5)	0.0051 (5)	0.0015 (5)
C26	0.0196 (6)	0.0251 (7)	0.0231 (6)	−0.0007 (5)	0.0017 (5)	0.0022 (5)
C211	0.0207 (6)	0.0224 (6)	0.0236 (6)	0.0015 (5)	0.0013 (5)	0.0025 (5)
O211	0.0464 (6)	0.0297 (5)	0.0260 (5)	0.0143 (5)	−0.0089 (4)	−0.0024 (4)
O212	0.0435 (6)	0.0210 (5)	0.0344 (6)	0.0046 (4)	−0.0092 (5)	−0.0014 (4)
N23	0.0331 (7)	0.0376 (7)	0.0257 (6)	−0.0022 (5)	−0.0035 (5)	0.0070 (5)
O231	0.0792 (10)	0.0407 (7)	0.0320 (6)	−0.0072 (6)	−0.0157 (6)	−0.0005 (5)
O232	0.0626 (8)	0.0452 (7)	0.0350 (6)	0.0073 (6)	−0.0147 (6)	0.0137 (5)
N25	0.0315 (6)	0.0240 (6)	0.0391 (7)	0.0038 (5)	0.0071 (5)	−0.0005 (5)
O251	0.0621 (8)	0.0255 (6)	0.0474 (7)	0.0155 (5)	0.0130 (6)	0.0065 (5)
O252	0.0563 (8)	0.0337 (6)	0.0447 (7)	0.0083 (5)	−0.0094 (6)	−0.0113 (5)
C31	0.0211 (6)	0.0228 (6)	0.0260 (7)	0.0041 (5)	0.0055 (5)	0.0020 (5)
C32	0.0223 (6)	0.0195 (6)	0.0291 (7)	0.0040 (5)	0.0036 (5)	0.0026 (5)
C33	0.0214 (6)	0.0249 (7)	0.0289 (7)	0.0055 (5)	0.0016 (5)	−0.0004 (5)
C34	0.0234 (7)	0.0297 (7)	0.0300 (7)	0.0076 (5)	0.0024 (5)	0.0070 (6)
C35	0.0221 (7)	0.0213 (7)	0.0393 (8)	0.0048 (5)	0.0061 (6)	0.0090 (6)
C36	0.0217 (6)	0.0229 (7)	0.0323 (7)	0.0022 (5)	0.0050 (5)	0.0005 (5)
C311	0.0226 (6)	0.0269 (7)	0.0255 (7)	0.0015 (5)	0.0049 (5)	0.0011 (5)
O311	0.0533 (7)	0.0325 (6)	0.0300 (6)	−0.0053 (5)	−0.0068 (5)	−0.0006 (4)
O312	0.0335 (6)	0.0264 (5)	0.0340 (6)	−0.0007 (4)	−0.0064 (4)	0.0083 (4)
N33	0.0312 (7)	0.0320 (7)	0.0327 (7)	0.0075 (5)	−0.0026 (5)	−0.0048 (5)
O331	0.0566 (8)	0.0308 (6)	0.0460 (7)	−0.0070 (5)	−0.0067 (6)	−0.0034 (5)
O332	0.0502 (7)	0.0450 (7)	0.0351 (6)	0.0144 (6)	−0.0146 (5)	−0.0037 (5)
N35	0.0291 (7)	0.0271 (7)	0.0525 (9)	0.0005 (5)	0.0003 (6)	0.0147 (6)
O351	0.0582 (8)	0.0542 (8)	0.0540 (8)	−0.0174 (6)	−0.0167 (6)	0.0333 (7)
O352	0.0638 (9)	0.0254 (6)	0.0670 (9)	−0.0057 (6)	0.0196 (7)	0.0031 (6)

### (Z)-3-(2-Chloro-9H-thioxanthen-9-yl)-N,N-dimethylpropan-1-aminium hydrogen bis(3,5-dinitrobenzoate) (II) Geometric parameters (Å, º)

**Table:**

N1—C112	1.4874 (18)	C112—H11E	0.9800
N1—C111	1.4905 (19)	C112—H11F	0.9800
N1—C1	1.4993 (16)	C21—C22	1.3862 (19)
N1—H1	0.867 (18)	C21—C26	1.3902 (19)
C1—C2	1.5245 (19)	C21—C211	1.5167 (18)
C1—H1A	0.9900	C22—C23	1.3879 (19)
C1—H1B	0.9900	C22—H22	0.9500
C2—C3	1.5096 (18)	C23—C24	1.382 (2)
C2—H2A	0.9900	C23—N23	1.4699 (18)
C2—H2B	0.9900	C24—C25	1.382 (2)
C3—C19	1.3390 (19)	C24—H24	0.9500
C3—H4	0.9500	C25—C26	1.3842 (19)
C11—C12	1.383 (2)	C25—N25	1.4771 (18)
C11—C18B	1.3973 (19)	C26—H26	0.9500
C11—H11	0.9500	C211—O212	1.2278 (17)
C12—C13	1.390 (2)	C211—O211	1.2684 (17)
C12—Cl12	1.7317 (15)	N23—O231	1.2173 (19)
C13—C14	1.379 (2)	N23—O232	1.2226 (17)
C13—H13	0.9500	N25—O252	1.2239 (18)
C14—C14A	1.393 (2)	N25—O251	1.2254 (17)
C14—H14	0.9500	C31—C36	1.3905 (19)
C14A—C18B	1.4034 (18)	C31—C32	1.3928 (19)
C14A—S10	1.7677 (14)	C31—C311	1.5054 (19)
C14B—C15	1.3986 (19)	C32—C33	1.3846 (19)
C14B—C18A	1.4013 (19)	C32—H32	0.9500
C14B—S10	1.7685 (15)	C33—C34	1.380 (2)
C15—C16	1.382 (2)	C33—N33	1.4735 (19)
C15—H15	0.9500	C34—C35	1.381 (2)
C16—C17	1.390 (2)	C34—H34	0.9500
C16—H16	0.9500	C35—C36	1.384 (2)
C17—C18	1.387 (2)	C35—N35	1.4697 (18)
C17—H17	0.9500	C36—H36	0.9500
C18—C18A	1.403 (2)	C311—O311	1.2195 (18)
C18—H18	0.9500	C311—O312	1.2968 (18)
C18A—C19	1.4901 (18)	O312—H312	1.04 (3)
C18B—C19	1.4847 (19)	N33—O331	1.2215 (18)
C111—H11A	0.9800	N33—O332	1.2249 (17)
C111—H11B	0.9800	N35—O352	1.222 (2)
C111—H11C	0.9800	N35—O351	1.2346 (19)
C112—H11D	0.9800		
			
C112—N1—C111	110.63 (12)	H11A—C111—H11C	109.5
C112—N1—C1	112.10 (11)	H11B—C111—H11C	109.5
C111—N1—C1	110.30 (11)	N1—C112—H11D	109.5
C112—N1—H1	110.5 (11)	N1—C112—H11E	109.5
C111—N1—H1	106.9 (11)	H11D—C112—H11E	109.5
C1—N1—H1	106.2 (11)	N1—C112—H11F	109.5
N1—C1—C2	113.84 (11)	H11D—C112—H11F	109.5
N1—C1—H1A	108.8	H11E—C112—H11F	109.5
C2—C1—H1A	108.8	C22—C21—C26	119.97 (12)
N1—C1—H1B	108.8	C22—C21—C211	119.78 (12)
C2—C1—H1B	108.8	C26—C21—C211	120.22 (12)
H1A—C1—H1B	107.7	C21—C22—C23	118.38 (13)
C3—C2—C1	108.34 (11)	C21—C22—H22	120.8
C3—C2—H2A	110.0	C23—C22—H22	120.8
C1—C2—H2A	110.0	C24—C23—C22	123.46 (13)
C3—C2—H2B	110.0	C24—C23—N23	118.42 (12)
C1—C2—H2B	110.0	C22—C23—N23	118.12 (13)
H2A—C2—H2B	108.4	C23—C24—C25	116.29 (12)
C19—C3—C2	127.90 (13)	C23—C24—H24	121.9
C19—C3—H4	116.1	C25—C24—H24	121.9
C2—C3—H4	116.1	C24—C25—C26	122.59 (13)
C12—C11—C18B	120.02 (13)	C24—C25—N25	118.95 (12)
C12—C11—H11	120.0	C26—C25—N25	118.45 (12)
C18B—C11—H11	120.0	C25—C26—C21	119.30 (12)
C11—C12—C13	121.47 (13)	C25—C26—H26	120.3
C11—C12—Cl12	119.77 (11)	C21—C26—H26	120.3
C13—C12—Cl12	118.76 (11)	O212—C211—O211	127.43 (13)
C14—C13—C12	118.81 (13)	O212—C211—C21	118.79 (12)
C14—C13—H13	120.6	O211—C211—C21	113.76 (12)
C12—C13—H13	120.6	C211—O211—H312	122.5 (12)
C13—C14—C14A	120.66 (13)	O231—N23—O232	123.75 (13)
C13—C14—H14	119.7	O231—N23—C23	118.57 (12)
C14A—C14—H14	119.7	O232—N23—C23	117.68 (13)
C14—C14A—C18B	120.45 (13)	O252—N25—O251	124.41 (13)
C14—C14A—S10	118.85 (11)	O252—N25—C25	117.92 (12)
C18B—C14A—S10	120.70 (11)	O251—N25—C25	117.67 (13)
C15—C14B—C18A	120.96 (13)	C36—C31—C32	120.02 (13)
C15—C14B—S10	118.13 (11)	C36—C31—C311	118.77 (13)
C18A—C14B—S10	120.87 (10)	C32—C31—C311	120.98 (12)
C16—C15—C14B	119.83 (14)	C33—C32—C31	118.92 (13)
C16—C15—H15	120.1	C33—C32—H32	120.5
C14B—C15—H15	120.1	C31—C32—H32	120.5
C15—C16—C17	120.05 (13)	C34—C33—C32	122.73 (13)
C15—C16—H16	120.0	C34—C33—N33	118.31 (13)
C17—C16—H16	120.0	C32—C33—N33	118.93 (13)
C18—C17—C16	120.18 (14)	C33—C34—C35	116.57 (13)
C18—C17—H17	119.9	C33—C34—H34	121.7
C16—C17—H17	119.9	C35—C34—H34	121.7
C17—C18—C18A	120.97 (14)	C34—C35—C36	123.23 (13)
C17—C18—H18	119.5	C34—C35—N35	117.87 (13)
C18A—C18—H18	119.5	C36—C35—N35	118.76 (14)
C14B—C18A—C18	117.97 (12)	C35—C36—C31	118.44 (13)
C14B—C18A—C19	119.87 (12)	C35—C36—H36	120.8
C18—C18A—C19	122.16 (12)	C31—C36—H36	120.8
C11—C18B—C14A	118.49 (13)	O311—C311—O312	125.60 (13)
C11—C18B—C19	121.32 (12)	O311—C311—C31	121.09 (13)
C14A—C18B—C19	120.08 (12)	O312—C311—C31	113.22 (12)
C3—C19—C18B	124.38 (12)	C311—O312—H312	113.3 (17)
C3—C19—C18A	120.64 (12)	O331—N33—O332	124.32 (14)
C18B—C19—C18A	114.98 (11)	O331—N33—C33	117.84 (12)
C14A—S10—C14B	99.81 (6)	O332—N33—C33	117.84 (13)
N1—C111—H11A	109.5	O352—N35—O351	124.48 (14)
N1—C111—H11B	109.5	O352—N35—C35	118.25 (14)
H11A—C111—H11B	109.5	O351—N35—C35	117.21 (14)
N1—C111—H11C	109.5		
			
C112—N1—C1—C2	69.34 (16)	C21—C22—C23—N23	178.42 (12)
C111—N1—C1—C2	−166.89 (12)	C22—C23—C24—C25	−0.3 (2)
N1—C1—C2—C3	170.47 (11)	N23—C23—C24—C25	−179.31 (12)
C1—C2—C3—C19	138.56 (15)	C23—C24—C25—C26	1.1 (2)
C18B—C11—C12—C13	0.3 (2)	C23—C24—C25—N25	−179.97 (12)
C18B—C11—C12—Cl12	−179.78 (10)	C24—C25—C26—C21	−1.0 (2)
C11—C12—C13—C14	−1.9 (2)	N25—C25—C26—C21	−179.94 (12)
Cl12—C12—C13—C14	178.18 (11)	C22—C21—C26—C25	0.08 (19)
C12—C13—C14—C14A	0.8 (2)	C211—C21—C26—C25	178.35 (12)
C13—C14—C14A—C18B	1.8 (2)	C22—C21—C211—O212	−1.10 (19)
C13—C14—C14A—S10	−178.31 (11)	C26—C21—C211—O212	−179.38 (13)
C18A—C14B—C15—C16	−1.1 (2)	C22—C21—C211—O211	177.41 (12)
S10—C14B—C15—C16	−178.89 (11)	C26—C21—C211—O211	−0.87 (18)
C14B—C15—C16—C17	−0.4 (2)	C24—C23—N23—O231	−170.63 (14)
C15—C16—C17—C18	0.9 (2)	C22—C23—N23—O231	10.3 (2)
C16—C17—C18—C18A	0.1 (2)	C24—C23—N23—O232	10.0 (2)
C15—C14B—C18A—C18	2.1 (2)	C22—C23—N23—O232	−169.06 (14)
S10—C14B—C18A—C18	179.79 (10)	C24—C25—N25—O252	176.56 (13)
C15—C14B—C18A—C19	−177.44 (12)	C26—C25—N25—O252	−4.5 (2)
S10—C14B—C18A—C19	0.28 (18)	C24—C25—N25—O251	−3.67 (19)
C17—C18—C18A—C14B	−1.6 (2)	C26—C25—N25—O251	175.28 (13)
C17—C18—C18A—C19	177.92 (13)	C36—C31—C32—C33	−2.00 (19)
C12—C11—C18B—C14A	2.32 (19)	C311—C31—C32—C33	172.38 (12)
C12—C11—C18B—C19	178.45 (12)	C31—C32—C33—C34	−0.7 (2)
C14—C14A—C18B—C11	−3.39 (19)	C31—C32—C33—N33	−178.60 (12)
S10—C14A—C18B—C11	176.76 (10)	C32—C33—C34—C35	1.9 (2)
C14—C14A—C18B—C19	−179.57 (12)	N33—C33—C34—C35	179.82 (12)
S10—C14A—C18B—C19	0.58 (17)	C33—C34—C35—C36	−0.5 (2)
C2—C3—C19—C18B	5.4 (2)	C33—C34—C35—N35	−176.22 (12)
C2—C3—C19—C18A	−173.53 (13)	C34—C35—C36—C31	−2.1 (2)
C11—C18B—C19—C3	45.7 (2)	N35—C35—C36—C31	173.65 (12)
C14A—C18B—C19—C3	−138.19 (14)	C32—C31—C36—C35	3.3 (2)
C11—C18B—C19—C18A	−135.27 (13)	C311—C31—C36—C35	−171.21 (12)
C14A—C18B—C19—C18A	40.80 (17)	C36—C31—C311—O311	−6.4 (2)
C14B—C18A—C19—C3	137.78 (14)	C32—C31—C311—O311	179.19 (13)
C18—C18A—C19—C3	−41.7 (2)	C36—C31—C311—O312	170.31 (12)
C14B—C18A—C19—C18B	−41.26 (17)	C32—C31—C311—O312	−4.14 (18)
C18—C18A—C19—C18B	139.25 (13)	C34—C33—N33—O331	−175.17 (14)
C14—C14A—S10—C14B	146.45 (11)	C32—C33—N33—O331	2.87 (19)
C18B—C14A—S10—C14B	−33.69 (12)	C34—C33—N33—O332	4.49 (19)
C15—C14B—S10—C14A	−148.97 (11)	C32—C33—N33—O332	−177.47 (13)
C18A—C14B—S10—C14A	33.25 (12)	C34—C35—N35—O352	−166.90 (14)
C26—C21—C22—C23	0.70 (19)	C36—C35—N35—O352	17.1 (2)
C211—C21—C22—C23	−177.59 (12)	C34—C35—N35—O351	15.6 (2)
C21—C22—C23—C24	−0.6 (2)	C36—C35—N35—O351	−160.41 (14)

### (Z)-3-(2-Chloro-9H-thioxanthen-9-yl)-N,N-dimethylpropan-1-aminium hydrogen bis(3,5-dinitrobenzoate) (II) Hydrogen-bond geometry (Å, º)

**Table:**

*D*—H···*A*	*D*—H	H···*A*	*D*···*A*	*D*—H···*A*
N1—H1···O212	0.866 (17)	2.046 (17)	2.7476 (16)	137.9 (15)
N1—H1···O311	0.866 (17)	2.485 (17)	2.9848 (16)	117.5 (14)
O312—H312···O211	1.04 (3)	1.41 (3)	2.4197 (15)	161 (3)
C1—H1*B*···O211^i^	0.99	2.36	3.2621 (18)	151
C14—H14···O232^ii^	0.95	2.41	3.2917 (19)	155
C18—H18···O231	0.95	2.53	3.4386 (19)	161

Symmetry codes: (i) *x*, −*y*+1/2, *z*−1/2; (ii) −*x*, *y*−1/2, −*z*+1/2.
